# Interactions between Sedentary and Physical Activity Patterns, Lean Mass, and Bone Density in Arab Men

**DOI:** 10.1155/2019/5917573

**Published:** 2019-10-14

**Authors:** Shaea Alkahtani, Khalid Aljaloud, Sobhy Yakout, Nasser M. Al-Daghri

**Affiliations:** ^1^Department of Exercise Physiology, College of Sports Science and Physical Activity, King Saud University, Riyadh, Saudi Arabia; ^2^Chair for Biomarkers of Chronic Diseases, College of Science, King Saud University, Riyadh, Saudi Arabia

## Abstract

The study examined the associations between physical activity and appendicular lean mass (ALM), fat mass, and bone mineral density (BMD) and examined the role of physical activity on these variables. The participants included 497 men (age: 32.2 ± 10.4 years and body mass index: 28.2 ± 5.4 kg/m^2^). The study was cross-sectional, and measurements included body composition measured on dual-energy X-ray absorptiometry and self-reported physical activity assessed using the International Physical Activity Questionnaire. Physical activity, including light physical activity, was associated with increased ALM (*P* ≤ 0.05). Fat indicators, including waist circumference, were positively associated with ALM (*P* ≤ 0.05), but not with BMD. Physical activity, including moderate-to-vigorous physical activity, was not associated with BMD, whereas ALM and handgrip strength were significantly associated with BMD (*P* ≤ 0.05). Physical activity independent of exercise intensity was directly associated with ALM and indirectly associated with BMD through increased muscle mass and strength.

## 1. Introduction

Sarcopenia is an age-related loss of muscle mass and strength and has been recognized as a muscle disease with an ICD-10-MC Diagnosis Code in 2016 [1]. The burden of sarcopenia on healthcare system is high because it increases the number of bone fracture injuries, the admission of hospitals, and the cost of care during hospitalization [2]. The European Working Group on Sarcopenia in Older People (EWGSOP) has recently released the second revised consensus on the definition and diagnosis of sarcopenia [2], and muscle strength has been elevated as the forefront characteristic of sarcopenia. It has suggested that low muscle strength which can be measured using handgrip strength through dynamometer is an indication of probable sarcopenia, and low muscle quantity or quality which can be reported as total appendicular lean mass is an indication of confirmed sarcopenia, and low physical performance which can be reported through various measures such as gait speed, the Short Physical Performance Battery, and the Timed-Up and Go test is an indication of severe sarcopenia [2]. Confirmed sarcopenia can be estimated by diving the appendicular lean mass (ALM) by the square of height in meters, which is usually measured using dual-energy X-ray absorptiometry (DXA). Confirmed sarcopenia is defined as two standard deviations below the mean value (2 SDs) of ALM in sex-specific young adults aged between 18 and 39 years, and a decrease of 1 standard deviation (1 SD) is considered as class I sarcopenia [3]. The loss of muscle mass begins at the age of 30 years, reaching its peak after the age of 50 years, whereas muscle strength reaches its maximal level at the age of 30 years to sustain until approximately the age of 50 years, when reduction starts [4]. Muscle loss in the lower extremities is greater than in other parts of the human body, which partially contributes to the increased incidence of falls and the sway in older individuals.

Modifiable factors such as physical activity and nutrition can reduce the acceleration of the sarcopenia process and prevent consequent frailty, falls, and fractures [5]. Accumulated evidence suggests that resistance training improves sarcopenia, bone density, functional status, and hip fracture [6]. Whether habitual and daily free-living physical activities impact muscle mass and strength is not fully known. Physical activity can be assessed using objective and subjective measures, both of which have been used in combination with sarcopenia indexes. The physical activities of 624 Australian adults aged > 60 years were assessed using a self-reported questionnaire. In the subjects, the prevalence of sarcopenia was 10.6% in men and 14.5% in women, and the sarcopenic individuals were less active than the nonsarcopenic individuals [7]. By using a self-reported physical activity questionnaire, a 5-year study found that the prevalence of sarcopenia at baseline was 7.3%, which increased to 16.8% after 5 years, of whom 14.8% were among the least active individuals [8]. The International Physical Activity Questionnaire (IPAQ) allows the inclusion of 4 self-reported domains, namely, activity work, leisure, transport, and domestic physical activity, and is valid for differentiating between age groups [6].

Measurements of fat mass and waist circumference (WC), muscle mass and strength, and bone mineral density (BMD) reflect overweight and obesity, sarcopenia, and osteoporosis, respectively. The coexistence of these impaired health aspects has been identified as osteosarcopenic obesity and has been proposed as a distinct entity [9]. Although age is considered the main external factor that influences sarcopenia, the role of fat content is also important. For example, among 1286 older British men aged > 70 years, 20% had sarcopenia, of whom 25% were obese [10]. ALM was positively associated with body fat among Korean women, and the odds ratio (OR) of sarcopenia for osteopenia and osteoporosis remained significant when adjusted for age but was not significant when adjusted for fat and physical activity [11]. The fact that this phenomenon has been observed in young healthy overweight/obese men and women age between 18 and 21 years is alarming [12]. Thus, early measure of sarcopenia and osteoporosis through measurement of muscle and bone contents is critical, particularly among overweight/obese individuals. The present study is aimed at investigating the interactions between physical activity, fat mass, and bone density on the indexes of sarcopenia.

## 2. Materials and Methods

### 2.1. Participant Characteristics

528 men expressed their interest in participation to the study, and 497 completed all the tests (age: 32.2 ± 10.4 years; body mass index (BMI): 28.2 ± 5.4 kg/m^2^; fat percentage: 31.5% ± 8.0%; BMD: 1.2 ± 0.1 g/cm^2^; and ALM: 8.8 ± 1.3 kg/ht^2^). They were from different areas but mostly from Riyadh and nearby cities such as Al-Kharj and Buraidah in Saudi Arabia, and some participants came from distant cities such as Mecca, Jeddah, and Al-Ahsa. Inclusion criterion was Arab men living in Saudi Arabia. The exclusion criterion was to have a diagnosed illness that affects muscle mass, balance, and/or ability to move, and people with medication for osteoporosis. Professional athletes were excluded, but recreational highly active people were accepted.

### 2.2. Study Procedure

The study was cross-sectional using a convenience sample and were conducted at College of Sport Sciences and Physical Activity at King Saud University (KSU), Riyadh, Saudi Arabia. All the participants who expressed their interest in the study received a full explanation of the study procedure and were instructed to arrive at the university in the morning before having breakfast. Written informed consent was provided by all the participants. Measures of the study included anthropometry (height, weight, and WC), handgrip strength test, ALM using DXA, and physical activity using the IPAQ. The study protocol was approved by the institutional review board (IRB) of KSU (IRB No. E-16-1785).

#### 2.2.1. 1st: Anthropometry

Height was measured to the nearest 0.1 cm using a stadiometer (Seca 213, seca GmbH & Co., Hamburg, Germany) while participant standing with back against the vertical stadiometer and feet together without shoes asking him to look straight ahead with head in the Frankfort horizontal plan, placing headboard on the scalp lightly. Body weight was measured to the nearest 0.1 kg, using a digital scale (PD100 ProDoc, Detecto Scale, Cardinal, Webb City, MO, USA), placing the scale on a hard flat surface and scale digital screen indicating zero, participants are standing calmly on both feet without shoes on the scale wearing light clothes and looking straight ahead for a few seconds until the reading appears on the scale screen. WC was measured at the umbilicus to the nearest 0.1 cm by using a measuring tape. The participants were instructed to exhale while standing, and the research assistant took 2 or 3 measurements of the waist.

#### 2.2.2. 2nd: Body Composition

The total body composition was measured using DXA (Lunar iDXA, GE Healthcare, General Electric Company, USA), following a standard operation procedure [13], and performed by a qualified technician. Body composition, including fat mass, total and appendicular lean mass, and BMD, were determined from the output. ALM was calculated by dividing the appendicular lean mass by the square of height in meters. The participants with −1 SD and −2 SDs were determined in accordance with the sex-specific means for Saudi young adults [14]. Fat mass index (FMI) was determined by dividing the total body fat mass by the square of height in meters.

#### 2.2.3. 3rd: Muscle Strength

Handgrip strength of the dominant hand was measured using a manual spring dynamometer (Baseline® Smedley Spring Dynamometers, Fabrication enterprises Inc., NY, USA); the handle was adjusted to a comfortable handgrip size for the participant who was instructed to squeeze the handle with maximal force while standing and with the elbow was fully extended. The better of two measures was recorded in kilograms [15, 16].

#### 2.2.4. 4th: Physical Activity

The paper version of the long-form IPAQ [17] was completed by all participants, with the presence of the research assistant, who explained the questionnaire and answered any questions.

### 2.3. Statistical Analyses

Data were analyzed using SPSS version 20 for Windows. The median value was used with data that were not normally distributed. The differences between the participants were examined using a *t* test when they were divided by the mean or median value and analysis of variance (ANOVA) when they were divided by quartile. Data were adjusted for age using covariate ANOVA. An *α* level of 0.05 was used to determine statistical significance, and post hoc analyses were conducted for significant interactions by using the Bonferroni correction.

## 3. Results

Descriptive data showed that 18.9% (*n* = 94) had a ALM of 1 SD below the mean value, and only 1.6% (*n* = 8) had a ALM of 2 SDs below the mean value. The mean handgrip strength was 42 kg, and only 4.3% (*n* = 22) of the participants had a handgrip strength of <30 kg. Of the subjects, 32.2% participated for >150 minutes per week in moderate-to-vigorous physical activity (MVPA); the characteristics of participants in the study variables are presented in Table 1.

Table 2 shows that the levels of the fat indicators decreased with the decrease in ALM based on the level mean value of classes I (−1 SD) and II (−2 SDs). Physical activity and physical strength significantly increased with the increase in ALM, but this was not found in sedentary time.  Table 3 shows the differences between physical activity levels and manual handgrip strength based on the mean value of ALM.

The study variables were divided into quartiles based on ALM, WC, and BMD, which reflect obesity, osteopenia, and sarcopenia, respectively. The study variable quartiles (Q1–Q4) based on ALM showed significant differences at ≤0.05 between Q1 and Q2 as compared with Q4 in terms of WC, fat percentage, and FMI.

When the study variables were divided into quartiles based on WC, ALM and BMD significantly increased with the increases in fat percentage and WC, with significant differences among all the quartiles (Q1, Q2, Q3, and Q4; *P* ≤ 0.05). For the physical activity variables, no significant differences in low physical activity (LPA), sedentary activity, and handgrip strength were found among the quartiles. Inverse associations were found between MVPA and WC and fat percentage, with significant differences between Q1 and the other quartiles of MVPA (*P* ≤ 0.05) based on the fat indicators.

**Figure 1:**
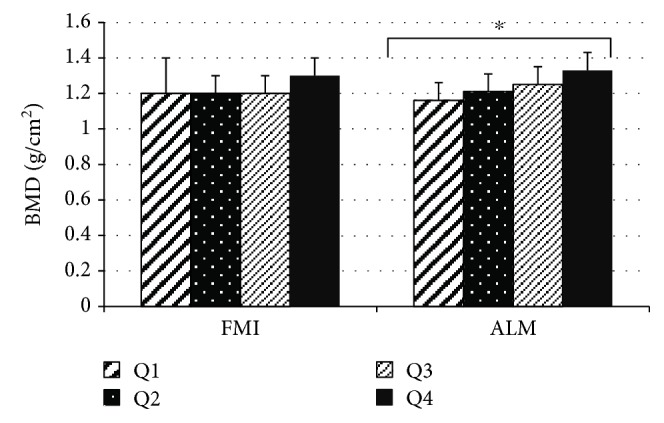
Differences among quartiles of appendicular lean mass (ALM) and fat mass index (FMI) based on bone mineral density (BMD). ^∗^Significant differences among all quartiles (Q1–Q4).

The quartiles of the study variables based on BMD showed no significant differences among the quartiles of MVPA, LPA, and sedentary activity, whereas the Q3 and Q4 of the handgrip strength were significantly higher than Q1 (*P* ≤ 0.05). Significant differences were found among all the ALM quartiles, but only Q4 for fat was significantly greater than Q2 and Q3 (*P* ≤ 0.05). No significant differences in FMI were found among the quartiles (Figure 1).

When adjustment for age was made, the relationships among the study variables were not affected.

## 4. Discussion

The present study is aimed at examining the association between ALM, fat mass, and BMD and at investigating the role of physical activity. The vast majority of participants did not have sarcopenia (−2 SD of the mean value) based on the Saudi population reference value [14] and only 1.6% can be identified as presarcopenic, and 18.9% were −1 SD below the mean value which can be considered as class I but are not classified as presarcopenic. Likewise, only 4.3% had a handgrip strength lower than 30 kg and are considered to have sarcopenia based on the previous criteria of EWGSOP established on 2010 [3], and only 2.6% of participants has a handgrip strength of ≤27 kg whom can be classified to have probable sarcopenia based on the recent revised criteria of EWGSOP2 [2]. It should be considered that the mean age was <35 years, such that age did not affect the current relationships between the variables. Physical activity was significantly associated with muscle mass and strength, whereas sedentary activity did not have any role effect. Increased WC, fat percentage, and FMI were associated with increased ALM. Compared with FMI, ALM had a significantly greater association with BMD. While MVPA had an inverse association with fat, only handgrip strength had an association with BMD. These data suggest the role of physical activity in ALM and the role of muscle mass presented as ALM and muscle strength in BMD. In addition, while fat had a positive association with ALM, it did not affect BMD.

Accumulated evidence suggests the role of physical activity in the prevention and/or delay of the onset of sarcopenia and causational impact of physical inactivity on sarcopenia and functional disability [18]. A recent systematic review and meta-analysis confirmed that habitual physical activity is protective against sarcopenia in middle-aged and old individuals [19]. The roles of MVPA in health, increased muscle mass, and the prevention of sarcopenia have been previously confirmed. For example, individuals with MVPA > 150 minutes per week had greater lean mass and lower limb strength [20]. High MVPA was associated with higher muscle mass and strength at baseline, and increased MVPA was associated with a lower incidence of sarcopenia (odds ratio (OR) = 0.64) in older adults [8]. However, the cutoff physical activity in terms of volume and intensity, which can positively affect lean mass and strength, has not been determined yet. A recent scientific report confirmed the absence of a cutoff physical activity level to attain health improvement and a minimal threshold for daily accumulated physical activity bouts, which suggest the role of shifting from sedentary to active lifestyle regardless of exercise intensity and acute exercise duration [21]. Objectively measured physical activity, including MVPA, LPA, sedentary activity, and breaks in sedentary behavior were independently associated with risk of severe sarcopenia in older British men [10]. A recent study showed a correlation between LPA and high density lipoprotein (HDL) [22].

While these studies and the scientific report confirmed the importance of LPA in reducing the incidences of cardiovascular risk factors, type 2 diabetes, and all-cause mortality, our present data showed a significant correlation between increased ALM and LPA. This could partially explain the mechanism of the role of LPA in health aspects. Improving strength and having greater muscle mass are important for increasing the engagement in aerobic physical activity [6], although some previous studies did not find a strong correlation between LPA and increased muscle mass. For example, the results of a multinomial logistic regression analysis showed that every 30 min of MVPA was associated with a reduced risk of severe sarcopenia (relative risk (RR): 0.53); sarcopenic obesity (RR: 0.47), LPA, and breaks in sedentary behavior were marginally associated with reduced sarcopenic obesity; and sedentary behavior was marginally associated with increased sarcopenic obesity independent of MVPA (RR: 1.18) [10].

Our results are in agreement with those of a study in young Australian adults aged between 19 and 22 years that found that lean body mass was strongly associated with total bone mineral density measured using DXA [23]. Unlike some studies, the present study did not find any adverse associations of increased fat mass with WC and BMD. A recent study found that increased obesity measured on the basis of fat content could be associated with decreased muscle and/or bone content, which reflect osteosarcopenic obesity [12]. Evidence shows that muscle mass is not normally distributed according to BMI categories and widely varies depending on the reference of muscle mass, particularly among individuals with low and high BMI. For example, the association concordance between FMI and BMI was examined among male and female adults, and women with higher FMI than BMI had low BMD than women with lower FMI than BMI; this suggests that increased body weight with increased fat mass is deleterious to the bone [24]. In a meta-analysis study using the data of 20,000 adults, the correlation between lean mass and femoral neck BMD was significantly greater than the similar correlation between fat mass and femoral neck BMD [25]. This may explain the association between physical activity that leads to increased muscle mass and BMD. An important finding by a longitudinal UK Biobank Study was that a strong adverse association existed between BMI and WC at baseline and follow-up MVPA levels, and a strong association between grip strength at baseline and follow-up MVPA levels, which confirms the role of body composition and muscle strength in promoting free-living physical activity [26].

The present data show no significant effect of age on the relationship between lean mass, fat mass, BMD, and physical activity. The current relationships are similar to those in elderly patients, except for the relationship between fat mass and BMD among women. For example, the effect of lean mass on BMD was greater in men and premenopausal women than in postmenopausal women [25]. The greater effect of fat mass than lean mass on BMD among postmenopausal women was previously demonstrated [27], and the absence of a correlation between lean mass and BMD was also found among middle-aged men with higher BMIs [28]. In men and women aged >50 years, increased lean mass was associated with BMD, although it did not affect femoral strength index, whereas increased fat mass did not affect BMD and had an adverse effect on femoral strength index [29].

The strength of the present study includes the fact that the participants came from a whole community and different suburbs, with few of them coming from different cities. The reference value of ALM for young Saudi men was used for the first time in this study as a specific population reference. Unlike those in many studies, all the participants in this study were assessed using the same DXA device. The limitations include its nature as a cross-sectional study recruiting a convenience sample whom might be enthusiastic and care of their health, which may not represent the whole community. Future studies are suggested to include women and with longitudinal series of measurements. Inclusion of known markers affecting musculoskeletal health such as irisin may also provide insights in understanding these bone and muscle cross-talk associations in the population [30].

## 5. Conclusion

In conclusion, physical activity independent of exercise intensity is effective in increased ALM, which partially affects BMD through muscle mass and strength. Although fat mass has a positive relationship with ALM, it was not associated with BMD.

## Figures and Tables

**Table 1:** Characteristics of the participants in the study variables.

Variable	% (*n*)
Normal weight (BMI < 25 kg/m^2^)	30.8 (153)
Overweight (BMI = 25 ≤ 30 kg/m^2^)	37.8 (188)
Obese (BMI ≥ 30 kg/m^2^)	31.3 (156)
WC (≥102 cm)	24.9 (124)
ALM (<1 SD)	18.9 (94)
ALM (<2 SD)	1.6 (8)
MVPA (≥150 min/week)	32.2 (161)
Sedentary behavior—sitting time (<6 hr per day)	8.4 (43)
Handgrip strength (≥30 kg)	4.3 (22)

Data expressed as percentage and number of participants. BMI: body mass index; WC: waist circumference; ALM: appendicular lean mass; MVPA: moderate-to-vigorous physical activity.

**Table 2:** Mean fat indices and BMD based on the ALM class categories.

Variables	ALM (kg/ht^2^)			
	≥8.97 (*n* = 220)	<8.97 (*n* = 277)	<7.74 (*n* = 94)	<6.51 (*n* = 8)
Waist circumference (cm)	99.4 ± 13.9^A^	87.3 ± 11.9	81.7 ± 11.2	70.9 ± 7.2
Fat (%)	33.2 ± 8.2^A^	30.1 ± 7.6	28.4 ± 7.9	22.6 ± 7.1
FMI (fat/ht^2^)	11.2 ± 2.8^A^	10.4 ± 2.8	9.9 ± 3.0	7.8 ± 2.5

Data are presented as mean ± SD. The superscript letter “A” refers to the statistical significance.

**Table 3:** Mean ALM (kg/ht^2^) according to levels of physical activity, sedentary activity, and handgrip strength.

Variable	Yes	No
MVPA (≥150 min/week)	9.1 ± 1.3^A^	8.7 ± 1.3
LPA (≥110 min/day)	9.0 ± 1.2^A^	8.7 ± 1.3
Sedentary behavior (≥6 hr per day)	8.8 ± 1.3	8.8 ± 1.1
Handgrip strength (≥42 kg)	9.2 ± 1.2^A^	8.4 ± 1.2
Handgrip strength (≥30 kg)	8.9 ± 1.3^A^	8.0 ± 1.1

Data are presented as mean ± SD. The superscript letter “A” refers to a statistical significance of ≤0.05. MVPA: moderate-to-vigorous physical activity, LPA: light physical activity.

## Data Availability

The data used to support the findings of the study are available from the corresponding author upon request.
